# Pre- and Postnatal Vitamin A Deficiency Impairs Motor Skills without a Consistent Effect on Trace Mineral Status in Young Mice

**DOI:** 10.3390/ijms251910806

**Published:** 2024-10-08

**Authors:** Joseph Arballo, Jennifer M. Rutkowsky, Marjorie J. Haskell, Kyla De Las Alas, Reina Engle-Stone, Xiaogu Du, Jon J. Ramsey, Peng Ji

**Affiliations:** 1 Department of Nutrition, University of California, Davis, CA 95616, USA; jarballo@ucdavis.edu (J.A.); mjhaskell@ucdavis.edu (M.J.H.); kdelasalas@ucdavis.edu (K.D.L.A.); renglestone@ucdavis.edu (R.E.-S.); xdu@ucdavis.edu (X.D.); 2 Department of Molecular Biosciences, University of California, Davis, CA 95616, USA; jrutkowsky@ucdavis.edu (J.M.R.); jramsey@ucdavis.edu (J.J.R.)

**Keywords:** maternal vitamin A deficiency, mouse model, motor dysfunction, growth retardation, concurrent micronutrient deficiencies

## Abstract

Pregnant women and children are vulnerable to vitamin A deficiency (VAD), which is often compounded by concurrent deficiencies in other micronutrients, particularly iron and zinc, in developing countries. The study investigated the effects of early-life VAD on motor and cognitive development and trace mineral status in a mouse model. C57BL/6J dams were fed either a vitamin A-adequate (VR) or -deficient (VD) diet across two consecutive gestations and lactations. Offspring from both gestations (G1 and G2) continued the same diets until 6 or 9 weeks of age. Behavioral assays were conducted to evaluate motor coordination, grip strength, spatial cognition, and anxiety. Hepatic trace minerals were analyzed. A VD diet depleted hepatic retinoids and reduced plasma retinol across all ages and gestations. Retracted rear legs and abnormal gait were the most common clinical manifestations observed in VD offspring from both gestations at 9 weeks. Poor performance on the Rotarod test further confirmed their motor dysfunction. VAD didn’t affect hemoglobin levels and had no consistent effect on hepatic trace mineral concentrations. These findings highlight the critical role of vitamin A in motor development. There was no clear evidence that VAD alters the risk of iron deficiency anemia or trace minerals.

## 1. Introduction

Vitamin A deficiency (VAD) is a prevalent micronutrient deficiency that disproportionally affects pregnant women and young children, posing a significant public health concern in low- and middle-income countries (LMICs) [1,2,3]. The World Health Organization estimates that approximately 250 million children worldwide suffer from preventable blindness caused by VAD [1,4]. A recent systematic review of population-based observational studies involving children and adolescents under 18 years from 40 LMICs reported a VAD (serum retinol < 0.7 µmol/L) prevalence of nearly 20% among children aged 0–5 years—the highest among all age groups [3]. Maternal vitamin A status is significantly associated with the risk of VAD in infants and children [5,6]. The transplacental transfer of retinol is a tightly regulated process that provides a constant amount of vitamin A to the fetus until the depletion of maternal stores [7,8,9]. Due to limited placental transfer, newborn infants, even those born to well-nourished mothers, have small hepatic retinol reserves [6]. Rapid postnatal growth heavily relies on the retention of vitamin A from maternal milk, which is estimated to contribute 60 times more to vitamin A stores than prenatal acquisition [6,10].

Vitamin A is indispensable for neural development. As a morphogen, retinoic acid (RA) is involved in regulating axial patterning and the formation of the neural plate and neural tube during embryogenesis. Severe VAD or excessive exposure to RA, particularly in early pregnancy, is teratogenic and can cause multiple congenital defects, such as malformed limbs, vertebral columns, and eyes, [11,12,13,14]. Additionally, RA promotes neurite growth and neuronal differentiation in vitro by binding to retinoid nuclear receptors (RAR or RXR) and transcriptionally regulating neuronal genes [15]. In a rodent model, dietary vitamin A deprivation in utero and up to 19 weeks of age led to progressive impairment of hippocampal CA1 long-term potentiation and long-term depression, both of which underlie learning and memory [16]. Restoration of vitamin A status in vivo or supplementation of retinoic acid in hippocampal tissue culture in vitro reinstated both forms of synaptic plasticity [16]. While the teratogenic effects, ocular manifestations, and impaired immunity caused by severe pre- and postnatal VAD have been extensively studied, there are still gaps in our understanding of the functional deficits in cognition and motor development, particularly in infants who are born without obvious birth defects but experience a gradual progression of VAD after birth. Clinical trials have yielded inconsistent findings regarding the impacts of postnatal VAD on neurocognitive and motor functions [17,18,19].

The challenge of VAD is compounded by concurrent deficiencies in other micronutrients, especially iron and zinc, which are even more prevalent in many developing countries [20,21,22]. Supplementing either or both iron and zinc increased plasma retinol concentration in a study of Mexican preschoolers [23], suggesting interactions between these trace minerals and vitamin A. Indeed, research from animal models has indicated both zinc and iron play roles in regulating the absorption, transport, mobilization, and metabolism of vitamin A [24,25,26,27]. However, fewer studies have examined the impact of VAD on zinc and iron status, although some evidence suggests that vitamin A intake affects zinc absorption in the small intestine and modulates iron metabolism and recycling by regulating hepcidin expression and erythrocyte clearance [28,29,30].

In this study, we used a mouse model to investigate functional impairments in cognition and motor skills resulting from pre- and postnatal VAD. For the first time, we assessed the developmental consequences of VAD across two consecutive gestations. Considering the high prevalence of multiple micronutrient deficiencies in LMICs, we further evaluated the effects of VAD on trace mineral status and the risk of anemia.

## 2. Results

### 2.1. Plasma and Hepatic Retinol Concentration

Our analysis did not indicate statistically significant differences in sex or the interaction between sex and treatment across the entirety of the study for applicable groups (Table 1). The plasma retinol concentration in G1 VD mice at 9 weeks of age was 0.031 µmol/L for males and below the detection limit for females, whereas G1 VR mice had an average of 0.61 µmol/L (Table 1). In G2 VD mice, plasma retinol concentrations were ≤ 0.045 and 0.02 µmol/L at 6 and 9 weeks of age, respectively, significantly lower than the corresponding values in aged-matched VR mice, which were 0.65 (average of males and females) and 0.75 µmol/L at 6 and 9 weeks, respectively. Across all VD mice, regardless of age and gestation, hepatic retinol concentrations were below the minimum detection limit. In contrast, hepatic retinol concentrations in VR mice were 0.27 µmol/g for G1 at 9 weeks, and 0.098 and 0.11 µmol/g for G2 at 6 and 9 weeks, respectively.

### 2.2. Clinical Manifestations

Mice were monitored for clinical signs of vitamin A deficiency (Table 2). Two G1 VD mice died at 9 weeks, and one G2 VD mouse died at 7 weeks. Ocular symptoms, such as milky-white opaque lenses resembling cataracts and corneal xerosis in humans, were observed in three G1 VD mice around 8 weeks of age. Additionally, nine VD mice (six in G1 and three in G2) exhibited constantly retracted rear legs and abnormal gait around 9 weeks of age. The overall incidence of clinical manifestations, including mortality, in the VD treatment was 63% for G1, and 0% and 50% for G2 6- and 9-week groups, respectively. In the VR treatment, these percentages were 5%, 0%, and 0%.

### 2.3. Growth and Development

**Figure 1:**
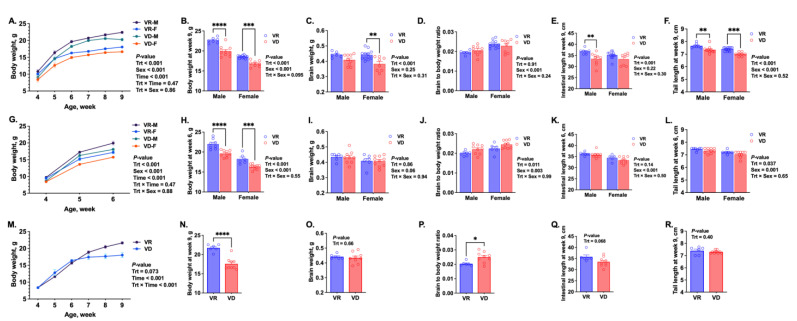
Effect of early-life vitamin A deficiency on growth and development of mouse offspring. The postweaning body weight change (**A**), body weight (**B**), brain weight (**C**), brain-to-body weight ratio (**D**), intestinal length (**E**), and tail length (**F**) of 9-week-old offspring born from the first gestation (G1, n = 6–12 mice/(sex∙treatment)). Figures (**G**–**L**) show the corresponding growth parameters of 6-week-old offspring born from the second gestation (G2, n = 6–10 mice/(sex∙treatment)), respectively. Figures (**M**–**R**) show the corresponding growth parameters of 9-week-old offspring born from the G2 (n = 6–9 mice/treatment), respectively. VR, vitamin A-adequate diet; VD, vitamin A-free diet. Data present LS means ± SEM. * *p* < 0.05, ** *p* < 0.01, *** *p* < 0.001, and **** *p* < 0.0001.

In the G1 groups, VD mice of both sexes exhibited lower growth rates that became more pronounced after weaning (Figure 1A,B, *P*_Trt_ < 0.0001). VAD significantly decreased brain weight at 9 weeks (Figure 1C, *P*_Trt_ = 0.0085), but the brain-to-body weight ratio (Figure 1D) did not differ between the two groups, suggesting symmetrical growth retardation. VD mice also had shorter intestinal length (Figure 1E, *P*_Trt_ = 0.0012) and shorter tails (Figure 1F, *P*_Trt_ < 0.0001). In the G2 6-week groups, VD impaired growth, resulting in lower body weights in both sexes at 6 weeks of age (Figure 1G,H, *P*_Trt_ < 0.001). However, VAD did not affect brain weight or intestinal length (Figure 1I,K), while it increased brain-to-body weight ratio and decreased tail length (Figure 1J,L, *P*_Trt_ ≤ 0.037). In the G2 9-week groups, VAD caused growth retardation in the last 3 weeks of the study (Figure 1M,N, *p* < 0.0001). Although the treatment did not affect tail length or brain weight (Figure 1O,R), it tended to decrease intestinal length (Figure 1Q, *p* = 0.0681). Consistent with the findings in the 6-week group, VAD led to a greater brain-to-body weight ratio at 9 weeks, indicating that brain development was better protected from VAD in the G2 mice compared to G1 offspring (Figure 1P, *p* = 0.0145). It is interesting that the adverse effect of VAD on brain growth, as measured by brain weight, was only observed in the G1 offspring, not in the G2.

### 2.4. Risk of Anemia and Trace Mineral Concentrations in the Liver

**Figure 2:**
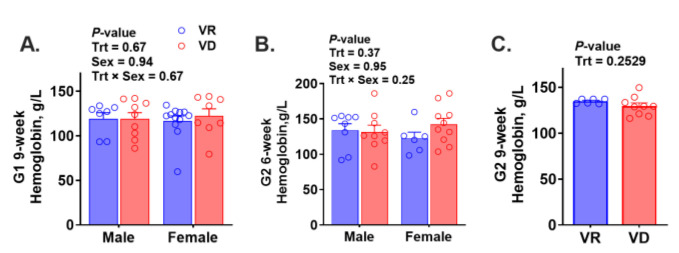
Effect of early-life vitamin A deficiency on blood hemoglobin concentration. Figures (**A**–**C**) show the hemoglobin concentrations in 9-week-old offspring born from the first gestation (G1, n = 7–12 mice/(sex∙treatment)) and 6-week (n = 6–10 mice/(sex∙treatment)) and 9-week-old offspring (n = 6–9 mice/treatment) born from the second gestation (G2), respectively. VR, vitamin A-adequate diet; VD, vitamin A-free diet. Data present LS means ± SEM.

**Figure 3:**
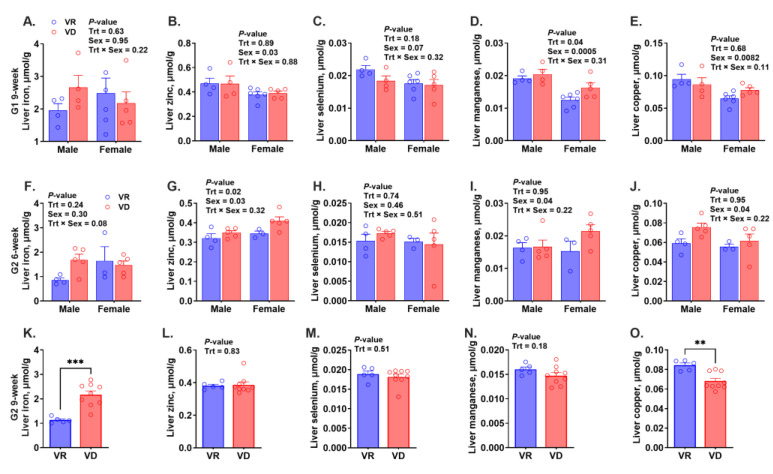
Effect of early-life vitamin A deficiency on hepatic trace mineral concentrations. Figures (**A**–**E**) show hepatic trace mineral concentrations in 9-week-old offspring born from the first gestation (G1, n = 7–12 mice/(sex∙treatment)). Figures (**F**–**J**) and figures (**K**–**O**) show hepatic trace mineral concentrations in 6-week-old (n = 6–10 mice/(sex∙treatment)) and 9-week-old (n = 6–9 mice/treatment) offspring born from the second gestation (G2), respectively. VR, vitamin A-adequate diet; VD, vitamin A-free diet. Data present LS means ± SEM. ** *p* < 0.01, and *** *p* < 0.001.

Hemoglobin (Hb) was analyzed to assess the presence of anemia (Figure 2A–C). No significant differences were observed between the two treatments, regardless of gestation or age. The mean Hb levels of all groups were above 110 g/L, indicating the absence of anemia, assuming the human cut-off value applies to rodents. Hepatic trace mineral concentrations are presented in Figure 3 A–O. Hepatic iron concentration was unaffected by treatment or sex in the G1 or G2 6-week groups, whereas VAD significantly increased hepatic iron levels in G2 9-week mice (*p* < 0.001). Vitamin A deficiency only altered hepatic zinc concentrations in the G2 6-week group regardless of sex (*P*_Trt_ = 0.02). Both treatment and sex significantly affected hepatic manganese concentration in G1 9-week-old mice (*P*_Trt_ = 0.049, *P*_sex_ < 0.001), with higher levels in male mice and within the VD group. Female mice from the G1 and G2 6-week groups had lower hepatic copper levels (*P*_sex_ ≤ 0.04), while VAD decreased hepatic copper levels only in G2 9-week-old mice (*p* = 0.003). Neither treatment nor sex significantly altered hepatic selenium concentration in any groups. In summary, there is no consistent treatment effect on hepatic trace mineral concentrations.

### 2.5. Motor Coordination

**Figure 4:**
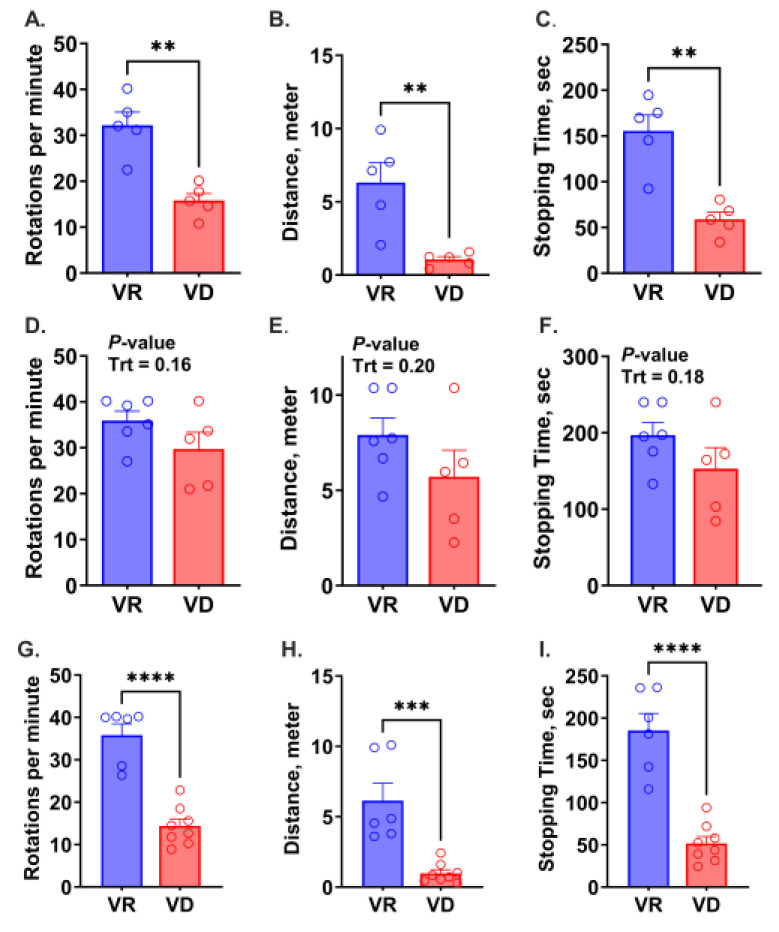
Effect of early-life vitamin A deficiency on motor function assessed through Rotarod test. Rotarod test performance of 9-week-old offspring born from the first gestation (G1, n = 5 mice/treatment) was assessed based on the highest speed of rotation (**A**), travel distance (**B**), and travel duration (**C**). Figures (**D**–**F**) and figure (**G**–**I**) show corresponding test parameters for 6- (n = 5–6/treatment) and 9-week-old (n = 6–8 mice/treatment) offspring born from the second gestation (G2), respectively. VR, vitamin A-adequate diet; VD, vitamin A-free diet. Data present LS means ± SEM. ** *p* < 0.01, *** *p* < 0.001, and **** *p* < 0.0001.

The observation of abnormal gait in VD mice led to an investigation of motor functions (Figure 4A–I). At 9 weeks of age, VR mice from both G1 and G2 outperformed their VD counterparts, exhibiting longer travel distances and durations, and higher rotating speed (*p* < 0.01). Although a similar trend was observed in 6-week-old G2 mice, none of the measurements were statistically significant (*p* > 0.1). The current study did not perform a statistical analysis of developmental (temporal) changes in motor coordination in G2 mice. However, a notable observation was the apparent deterioration in Rotarod performance in G2 VD mice from 6 to 9 weeks of age, indicating a progressive decline in performance with prolonged exposure.

### 2.6. Grip Strength

A grip strength test was used to assess the maximal muscle strength and neuromuscular function of limbs. VAD did not affect 2- or 4-limb grip strength in G1 mice or G2 6-week-old mice (Supplementary Figure S1). However, at 9 weeks of age, G2 VD mice exhibited greater 4-limb grip strength compared to VR mice (*p* = 0.004), but their 2-limb grip strength remained similar.

### 2.7. Anxiety Behavior

Treatment did not affect the time (% of total) spent in the open arms in either the G1 9-week group (n = 7–12 per sex∙treatment) or the G2 6-week group (n = 6–10 per sex∙treatment, Supplementary Figure S2), indicating no difference in the preference to explore or hide. However, male G1 VR mice made more entries into the closed arms than their VD counterparts, contributing to a significant interaction effect (*P* _Trt×sex_ = 0.02). There is no clear evidence suggesting a difference in anxiety behavior between treatments, while the large individual variations in the EPM test necessitate future analysis with a greater sample size.

### 2.8. Spatial Cognition

Spatial learning and memory were assessed in the G2 6-week group (n = 6–11 per sex∙treatment) using the BM test (Supplementary Figure S3). The latency to find the escape hole decreased with the increase in the number of trials (*P* _trial_ < 0.01), while neither treatment nor sex had a significant impact on this parameter (Supplementary Figure S4A). This finding suggests that all mice displayed a similar rate of task acquisition. On the probe day, none of the parameters was affected by treatment or sex, indicating no discernible differences in spatial cognition among the treatment groups (Supplementary Figure S4B–E).

## 3. Discussion

### 3.1. Developmental Outcomes and Clinical Symptoms

This study used a mouse model to investigate the developmental outcomes and hepatic trace mineral status in response to early-life VAD. Maternal milk is a rich source of vitamin A, providing the majority of retinol stores in suckling rodent pups, which can sustain their growth and health for an extended period before depletion [6,10]. Previous studies have shown that weaned rats did not exhibit significant reductions in plasma retinol concentrations until 24 weeks after being fed a vitamin A-free diet [31,32]. Laboratory mice demonstrate even greater resistance to dietary vitamin A deprivation, with symptoms of deficiency appearing only after 6–10 months of exposure [33,34]. To induce VAD in their offspring, it is crucial to significantly deplete maternal retinol reserves. Therefore, in this study, dams were fed the VD diet for two consecutive gestations and lactations. We anticipated that offspring born from the second gestation would be more susceptible to VAD than those of the first litter due to the further depletion of maternal stores. Growth retardation, a well-established sign of VAD, was observed in offspring from both gestations regardless of age. However, it is notable that G1 offspring were more vulnerable to VAD, exhibiting more severe growth and developmental impairments—such as shorter intestinal length and tail length, lower brain weight, and a higher number of clinical symptoms—compared to both G2 groups. Few studies have assessed the risk of VAD across multiple gestations, but evidence from a human study in Brazil suggests that milk retinol concentration is lower in primiparous mothers compared to multiparous mothers [35]. This feature of lactation biology may explain the greater vulnerability observed in G1 offspring if similar mechanisms apply to rodents.

### 3.2. Motor Incoordination

Ocular diseases are typical clinical symptoms of severe vitamin A deficiency, which are extensively reported in both clinical trials and translational research. However, in this study, motor anomalies are more prevalent than ocular manifestations. The abnormal gait and constant contraction of rear limbs observed in 9-week-old VD mice from both gestations indicated neuromuscular disorders. This was further supported by their poor performance in the Rotarod test, underscoring deficits in motor coordination, which was only detected at 9 weeks of age but not statistically significant in 6-week-old VD mice. A similar defect in hindlimbs was also observed in young adult rats after 6 months of feeding on a vitamin A-free diet [31]. The limb muscles are innervated by axons from motor neurons in the lateral motor column (LMC) that spans the brachial and lumber levels of the spinal cord [36,37,38]. Studies have shown that retinoid signaling is not only crucial for directing migration patterns and specifying subtype identity of LMC motor neurons during development [39] but also indispensable to the survival of motor neurons in adulthood [31,40]. Retinoid deficiency resulted in the neurodegeneration of motor neurons in the lumber cord of adult rats, manifesting increased neurofilament, neuronal vacuolar lesions, and astrogliosis [31]. The neuropathology was accompanied by motor incoordination resembling what was observed in the young mouse model in the current study.

Cellular RA is locally synthesized by retinaldehyde dehydrogenases (RALDHs) from retinal. However, unlike most LMC motor neurons, a specific subtype that innervates all intrinsic and certain extrinsic digit muscles was found to lack RALDH, particularly the encoding gene, Aldh1a2 [41]. This observation suggests that the development of digit-innervating motor neurons is independent of retinoic acid signaling. Extrinsic digit muscles, located in the forearms and lower legs, and intrinsic digit muscles, found in the hands and feet, both control digit movement and may influence grip strength. Therefore, the findings from that study provide a plausible molecular explanation for the unaffected grip strength observed in VD mice, despite their motor incoordination.

Although motor functions are not frequently assessed in clinical trials investigating VAD, a few human studies have reported mixed results on the impact of VAD on motor and cognitive development. In a randomized trial involving HIV-positive Tanzanian women, vitamin A supplementation during gestation and postpartum did not improve mental or motor functions in their offspring at 6 months of age [42]. Similarly, maternal vitamin A supplementation during pregnancy and lactation in undernourished communities in Nepal showed no significant effects on the motor and cognitive development of their children at 10 to 13 years of age [17]. In contrast, an oral vitamin A supplement given during infancy was associated with a modest improvement in psychomotor scores in Indonesian children at 3 years of age [19]. These varied outcomes from clinical trials underscore the complexity of the relationship between VAD and motor development, suggesting that contextual factors, such as nutritional status and population characteristics, may play a crucial role in determining the impact of vitamin A supplementation on motor development.

It is believed that the vital role of vitamin A in neurodevelopment and modulating neuronal function is carried out by RA which binds to its cognate nuclear receptors (RARs and RXRs) and subsequently regulates RA-response element-containing genes in the central nervous system [36]. Previous studies have demonstrated that dietary vitamin A deficiency or RAR-β knockout diminished or abolished long-term potentiation and long-term depression in hippocampal CA1, consequently impairing spatial cognition in adult mouse models [16,43]. However, neither spatial learning nor anxiety behaviors assessed at 6 weeks, nor anxiety assessed at 9 weeks of age, were significantly affected by feeding a vitamin A deficient diet in the current study.

### 3.3. Trace Mineral Status and Risk of Anemia

The high prevalence of concurrent micronutrient deficiencies in LMICs, particularly vitamin A, iron, and zinc, propagates the investigation of the interplay between these micronutrients. VAD has been shown to alter iron metabolism and modulate erythropoiesis and hemoglobin synthesis through mechanisms that are not completely understood [44,45,46,47,48]. In rat models, VAD has been associated with iron accumulation in the spleen and liver [30,49,50] and an increased risk of anemia [51]. Conversely, restoring vitamin A levels has been shown to promote recovery from iron deficiency (ID) [52], suggesting VAD impairs iron mobilization from body stores. However, human clinical trials have reported inconsistent effects of vitamin A supplementation on anemia and ID. A recent meta-analysis revealed that vitamin A supplementation increased hemoglobin levels in 15 out of 21 studies, with no effect observed in the remaining 6 studies [48]. Although supplementation generally reduced anemia prevalence (10 out of 11 studies), results were mixed regarding its impact on ID [48]. These findings imply that VAD may raise the risk of anemia through mechanisms independent of iron status.

In the current study, the VD diet neither affected Hb compared to the VR groups nor increased the risk of anemia (Hb < 110 g/L) in any age or gestation group based on the human cut-off value. Interestingly, VAD only increased hepatic iron accumulation in G2 mice at 9 weeks, but not in other groups. Overall, this study did not find conclusive evidence linking VAD to impaired iron mobilization or a heightened risk of anemia. Likewise, no evidence was found that VAD affects hepatic stores of other trace minerals. Future research should explore the impact of VAD on circulating trace mineral levels to provide a more comprehensive understanding.

### 3.4. Conclusions

This study, using a mouse model, demonstrates that prolonged dietary VAD during pregnancy and postnatal development results in significant growth retardation, ocular diseases, and impaired motor coordination. The motor deficits observed may represent a more prevalent clinical manifestation of VAD than ocular complications, highlighting the need for further research. The smaller sample size in the 9-week G2 group limited our ability to analyze the impact of sex in this cohort. Additionally, our findings do not support the notion that VAD impairs iron mobilization or alters the status of other trace minerals. However, further analyses are required to examine the effects of VAD on trace mineral metabolism, as this cannot be concluded from the current study. This study also suggests an increased susceptibility to VAD in offspring from the first parity, which warrants further investigation.

## 4. Materials and Methods

### 4.1. Experimental Design

All procedures in the animal experiments were reviewed and approved by the Institutional Animal Care and Use Committee of the University of California, Davis (Protocol# 22722, approval/expiration dates: 03-17-2022/03-17-2025). Eleven C57BL/6J female mice (8–9 weeks of age) were enrolled in the study and randomly assigned to dietary treatments, consisting of a vitamin A-adequate diet (4000 IU/kg, VR, n = 6) and a vitamin A-deprived diet (VD, n = 5), starting from the first breeding through to the end of the second lactation. The ingredient and nutrient compositions of both diets (Dyets Inc., Bethlehem, PA, USA) are provided in Supplementary Table S1. Mouse pups born from the first (G1) and second gestation (G2) continued on the same diet as their dams from weaning on postnatal day (PD) 21 until 9 weeks of age (G1 pups) or until 6 or 9 weeks of age (G2 pups). Diets were refreshed three times a week. After weaning, mouse pups were group-housed (2–4 mice per cage). Birth outcomes, including litter size, live birth, and congenital defects, were recorded on PD 5. Health and clinical signs of vitamin A deficiency (e.g., microphthalmia, ocular manifestations, mortality, motor anomalies) were monitored every other day after weaning. The body weight of the pups was recorded at weaning (PD21) and then weekly thereafter.

G1 pups were assessed at 9 weeks of age for anxiety using the elevated plus maze (EPM) test, grip strength, and motor coordination using the Rotarod test. G2 pups were assessed for anxiety and spatial cognition using the Barnes maze (BM) test, grip strength, and motor coordination at 6 weeks of age. G2 9-week-old pups underwent the same battery of behavioral tests as G1 pups, except they were not tested for anxiety. Both grip strength and Rotarod tests were performed on the same subset of mice that were randomly selected from each age and gestation group (n = 5–8 per sex∙treatment). G1 pups were euthanized on PD 63, and subgroups of G2 pups were euthanized on PD 42 and PD 63, respectively. Blood samples were collected in a sodium heparin vacutainer after decapitation under anesthesia. Plasma was harvested after centrifugation (1500× *g*, 4 °C, 15 min). Tissues were sampled and snap-frozen in liquid nitrogen. Brain weight as well as intestine and tail lengths were recorded.

### 4.2. Elevated Plus Maze Test for Anxiety

The elevated plus maze was conducted as previously reported with the following modifications [53]. Given rodents’ natural preference for darkness, the EPM test assesses anxiety-related behaviors by measuring activity (duration and entries) in the open and closed arms. The EPM apparatus consists of two open arms (30 cm L × 6 cm W × 1 cm H) and two closed arms (30 cm L × 6 cm W × 20 cm H), connected by a central platform (6 cm×6 cm) to form a cross. The maze is elevated above the floor. Naïve mice were acclimated to the test room for 1 h before the procedure. The test began by placing each mouse facing the edge of the same closed arm. Mice were allowed to explore the maze for 5 min. The test was conducted under white noise to reduce stress. Behavior was recorded by a camera mounted above the center of the maze, and the mice were tracked in real time using EthoVision XT 15 software (Noldus, Wageningen, Netherlands). The number of entries and the duration of time spent in the open and closed arms were recorded. The maze was cleaned with 70% ethanol between mice.

### 4.3. Barnes Maze Test for Spatial Cognition

The Barnes maze was conducted as previously reported with the following modifications [53]. The BM is a white acrylic circular platform with 20 evenly spaced holes along the edge. Only one hole (the escape hole) was connected to a black plastic box underneath the platform designed for the mouse to escape and hide. Test mice are expected to learn and use the three signs on the surrounding walls as spatial cues to locate the escape hole. Light (700 lux) was set to evenly illuminate the maze during the trials. On the first day of the four-day trial, mice were acclimated to the maze and guided to the escape hole twice with a translucent cylinder. The acclimation was followed by a single trial where mice were allowed to explore the maze until they discovered the location of the escape hole or the end of 180 s, whichever occurred first. Mice underwent two and three trials on days 2 and 3, respectively. If a mouse failed to locate the escape hole within 180 s during the trial days, it was guided to the escape hole with the cylinder. Mice were allowed to rest in the escape box with the lights off for 30 s before being removed from the apparatus. On day 4, the probe day, the box under the escape hole was removed, and mice were allowed to explore the apparatus for a single 180-s trial. The apparatus was cleaned with 70% ethanol between trials. Mice behaviors were analyzed using EthoVision software.

### 4.4. Grip Strength Test

The grip-strength test was conducted as previously reported with the following modifications [53]. The grip strength of the forelimbs and all four limbs was measured using a grip strength apparatus (Ametek test, Berwyn, PA, USA) equipped with a wire mesh push-pull scale that measures pulling force. Mice were positioned to grasp the wire with either forepaws or all paws and were then gently pulled back horizontally by the base of the tail at a steady, slow pace. The pulling force at the moment the mouse released the grip was recorded by the equipment. The test was repeated three times, followed by a 30-min break in the home cage, and then repeated for another three trials. Mice were allowed minimal rest (<30 s) between tests. Body weight was recorded before the test to normalize grip strength. The maximum grip strength from the six trials was used for analysis.

### 4.5. Rotarod Test for Motor Function

The RotaRod test was conducted as previously reported with the following modifications [53]. Motor coordination and balance were assessed using the Rotarod test (IITC Life Science, Woodland Hills, CA, USA). The test was set for 240 s, with a starting speed of 4 rotations per min (RPM), a maximum speed of 40 RPM, and an acceleration phase of 180 s to reach the maximum speed. Mice were placed on the Rotarod drum for 30 s to balance and acclimate before the rotation began. The rotating speed, travel distance, and duration were automatically recorded by the equipment when the mice either fell off the drum or completed the trial. The test was repeated 3 times, with a 20-min break in home cages between trials.

### 4.6. Plasma and Liver Retinol Analysis

Plasma and liver retinol concentrations were measured using reverse-phase HPLC (1260 Infinity II, Agilent Technologies, CA, USA) as described by previous studies [54,55]. A pooled plasma sample (UTAK Defibrinated Plasma, Valencia, CA, USA) was calibrated for retinol concentration using a control serum with a certified retinol concentration (SRM, 968f, NIST, Gaithersburg, MD, USA). Three aliquots of the pooled plasma were analyzed with each batch of plasma or liver samples to assess measurement precision. Liver retinol was analyzed following the method described by Handelman et. al. [55] with minor modifications. Briefly, 25 mg of liver tissue was homogenized and transferred to a glass vial containing 150 µL of 10% pyrogallol in ethanol and 1.5 mL of ethanol. The liver samples were then saponified for 1 h at 60 °C. Dl-Tocol (Cayman Chemical, Ann Arbor, MI, USA) was added as an internal standard (0.5 mL, 200 µg/mL). For plasma retinol analysis, 100 uL of plasma was mixed with 900 uL of HPLC-grade water in glass vials, followed by the addition of 1 mL of ethanol containing approximately 100 ng/mL of retinyl acetate as an internal standard. Retinol from hepatic and plasma samples was extracted into 3 mL of hexane by vigorous mixing for 2 min using an automated shaker. The samples were then centrifuged for 2 min to separate hexane from the polar layer at the bottom. Hexane layers were transferred to clean glass vials and dried under nitrogen gas. The dried samples were redissolved in 70 uL of methanol and vortexed for 15 s. To quantify retinol concentrations in plasma and liver samples, a ratio was derived from the retinol concentration of the sample divided by the internal standard concentration. The same was conducted for standardized human pooled plasma samples, which were averaged into a mean ratio. There, the ratio of individual samples was divided by the mean ratio of the human pooled plasma samples and then multiplied by the average retinol concentration of the human pooled plasma samples to calculate retinol concentration per sample. Retinol concentrations were measured using an HC-C18 column (4.6 × 150 mm, 5 µm) and an HC-C18 guard column (4.6 × 12.5 mm, 5 µm) (Agilent Technologies, Santa Clara, CA, USA). The mobile phase consisted of 75% of a mixture of acetonitrile and methanol (85/15 *v/v)* containing 0.01% ammonium acetate (*w*/*v*) and 25% 2-propanol, with a flow rate of 1 mL/min and a total run time of 10 min. The within-batch CV for the pooled plasma retinol analyses was less than 3% for plasma samples and less than 2% for liver samples. All chemicals were of HPLC grade, and all procedures were conducted under dim yellow light to prevent retinoid degradation.

### 4.7. Trace Mineral Analysis

Liver samples (~100 mg) were digested in 5 mL of 16 mol/L nitric acid (Trace metal grade, Fischer Scientific, Hampton, NH, USA) for at least 48 h. The samples were then heated on a heating plate until nearly all of the acid evaporated. The remaining residue was resuspended in ultrapure water (Milli-Q, MilliporeSigma, Burlington, MA, USA) and brought to a final volume of 5 mL in a volumetric flask. Trace minerals, including iron, zinc, copper, selenium, and manganese, were quantified through inductively coupled plasma optical emission spectroscopy (iCAP 6000, Thermo Scientific, Waltham, MA, USA) following a standard curve method. The mineral content in the 5-mL solution was calculated based on the analyzed concentration. To determine the mineral concentration in the wet tissue, the mineral content was divided by the wet tissue weight and the mineral molecular weight. Results of mineral concentrations were reported as µmol/g wet tissue.

### 4.8. Statistical Analysis

Data were analyzed using GraphPad Prism (v.10, San Diego, CA, USA). Normality was assessed using QQ-plot. A two- or three-way ANOVA with repeated measures was employed, where appropriate, to analyze the main effects of treatment, sex, time, and their interactions. An unpaired *t*-test was used when treatment was the only factor in the model. Due to the smaller sample size in the G2 9-week group, sex was not included in the statistical analysis for this group. All data were presented as means and standard errors.

## Figures and Tables

**Table 1:** Effect of vitamin A deficiency on plasma and liver retinol concentration.

Gestation, Week	Sex	Trt	Plasma Retinol, µmol/L	*p*-Value Plasma			Liver Retinol, µmol/g Tissue	*p*-Value Liver		
				Trt	Sex	Trt × Sex		Trt	Sex	Trt × Sex
G1, week 9	Male	VR	0.687 ± 0.099	<0.001	0.12	0.35	0.289 ± 0.114	0.0002	0.8268	0.8268
		VD	0.031 ± 0.031				N.D.			
	Female	VR	0.569 ± 0.024				0.264 ± 0.057			
		VD	N.D.				N.D.			
G2, week 6	Male	VR	0.649 ± 0.089	<0.001	0.85	0.89	0.101 ± 0.020	0.0001	0.7921	0.7921
		VD	0.030 ± 0.003				N.D.			
	Female	VR	0.651 ± 0.017				0.096 ± 0.009			
		VD	0.045 ± 0.007				N.D.			
G2, week 9	Pooled	VR	0.746 ± 0.062	<0.001			0.115 ± 0.033	0.0004		
		VD	0.020 ± 0.005				N.D.			

Data present LS means ± SEM. Trt, treatment; G1, first gestation; G2, second gestation; VR, vitamin A-adequate diet; VD, vitamin A-free diet; N.D. = not detectable (0.001 was imputed for statistical analysis).

**Table 2:** Effect of vitamin A deficiency on clinical manifestations.

	G1, 9 Week		G2, 6 Week		G2, 9 Week	
Symptoms and Mortality	VR	VD	VR	VD	VR	VD
Microphthalmia, n	1	1	0	0	0	1
Ocular symptom, n	0	3	0	0	0	0
Mortality, n	0	2	0	0	0	1
Motor anomaly, n	0	6	0	0	0	3
Total, n	1	12	0	0	0	5
Risk, % of total animals	5.2%	63.2%	0%	0%	0	50%

G1, first gestation; G2, second gestation; VR, vitamin A-adequate diet; VD, vitamin A-free diet.

## Data Availability

All data have been reported in this paper.

## Supplementary Materials

The following supporting information can be downloaded at https://www.mdpi.com/article/10.3390/ijms251910806/s1.
