# Supplementary material for: Pre- and Postnatal Vitamin A Deficiency Impairs Motor Skills without a Consistent Effect on Trace Mineral Status in Young Mice
Source: Int J Mol Sci. 2024 Oct 8;25(19):10806. doi: 10.3390/ijms251910806 (PMC11477164; doi:10.3390/ijms251910806)
Supplement: Supplementary file 1 [file ijms-25-10806-s001.zip › ijms-3189601-supplementary.pdf]

Pre- and postnatal vitamin A deficiency impairs motor skills without affecting trace mineral status in young mice

Table S1: Ingredient composition of experimental diets

	Vitamin A-adequate diet (DYET#119343)[a]	Vitamin A-free diet (DYET#119342)[a]
Ingredients	g/kg	g/kg
Vitamin free casein	200	200
L-cystine	3	3
Sucrose	94	98
Cornstarch	397.5	397.5
Dyetrose	132	132
Cottonseed oil with tBHQ	70	70
Microcrystallline cellulose	50	50
Mineral-vitamin premix (no vitamin A)[b]	45.04	45.04
Vitamin A palmitate	4	0
Choline bitartrate	2.5	2.5
Total	1000	1000

[a.] Diets were manufactured by Dyets Inc (Bethlehem, PA).

[b.] See Supplemental Table 2 for composition.

Pre- and postnatal vitamin A deficiency impairs motor skills without affecting trace mineral status in young mice

Table S2: Composition of mineral-vitamin premix[a]

Mineral compound	Amount in diet, mg/kg	Vitamin compound	Amount in diet, mg/kg
Calcium carbonate	12,495	Niacin	30.0
Potassium phosphate (monobasic)	6,860	Calcium pantothenate	16.0
Potassium citrate·H_2O	2,477.3	Pyridoxine HCl	7.0
Sodium chloride	2,590	Thiamine HCl	6.0
Potassium sulfate	1,631	Riboflavin	6.0
Magnesium oxide	840	Folic acid	2.0
Ferric citrate	43.9	Biotin	0.2
Zinc carbonate	57.75	Vitamin E acetate (500 IU/g)	150.0
Manganese carbonate	22.05	Vitamin B12 (0.1%)	25.0
Cupric carbonate	10.5	Vitamin A palmitate (500000 IU/g)	0.0
Potassium iodate	0.35	Vitamin D3 (400000 IU/g)	2.5
Sodium selenate	0.36	Vitamin K1/Dextrose mix (10 mg/g)	75.0
Ammonium paramolybdate·$4H_2O$	0.28		
Sodium metasilicate·$9H_2O$	50.75		
Chromium potassium sulfate·$12H_2O$	9.63		
lithium chloride	0.61		
Boric acid	2.85		
Sodium fluoride	2.22		
Nickel carbonate	1.11		
Ammonium vanadate	0.23		

[a.] Sucrose powder is used as carrier in the premix and makes the remaining amount of premix in the diet.

Pre- and postnatal vitamin A deficiency impairs motor skills without affecting trace mineral status in young mice

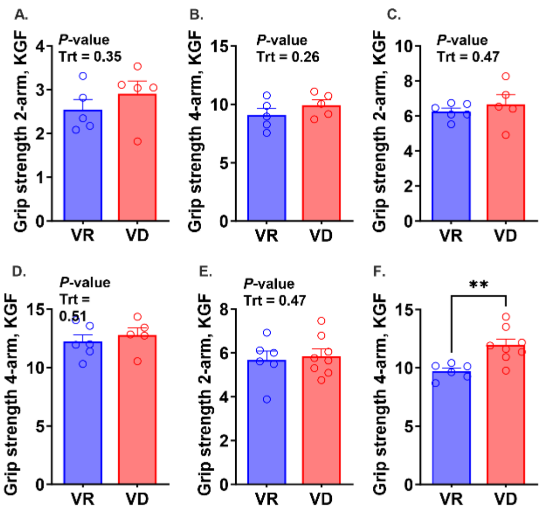

Figure S1. Effect of dietary vitamin A deficiency on grip strength of offspring normalized by body weight. The 1st gestation offspring (G1, N=5 per treatment) underwent testing for 2-arm grip strength (A) and whole-body grip strength (B). The 2nd gestation (G2, 6 wk, N= 5-6 per treatment) offspring underwent testing for 2-arm grip strength (C) and whole-body grip strength (D). The 2nd gestation (G2, 9 wk, N= 6-8 per treatment) offspring underwent testing for 2-arm grip strength (E) and whole-body grip strength (F). VR, vitamin A-adequate diet; VD, vitamin A-free diet. Data present LS means ± SEM. ∗ $P < 0.05$, ∗∗ $P < 0.01$, ∗∗∗ $P < 0.001$, and ∗∗∗∗ $P < 0.0001$.

Pre- and postnatal vitamin A deficiency impairs motor skills without affecting trace mineral status in young mice

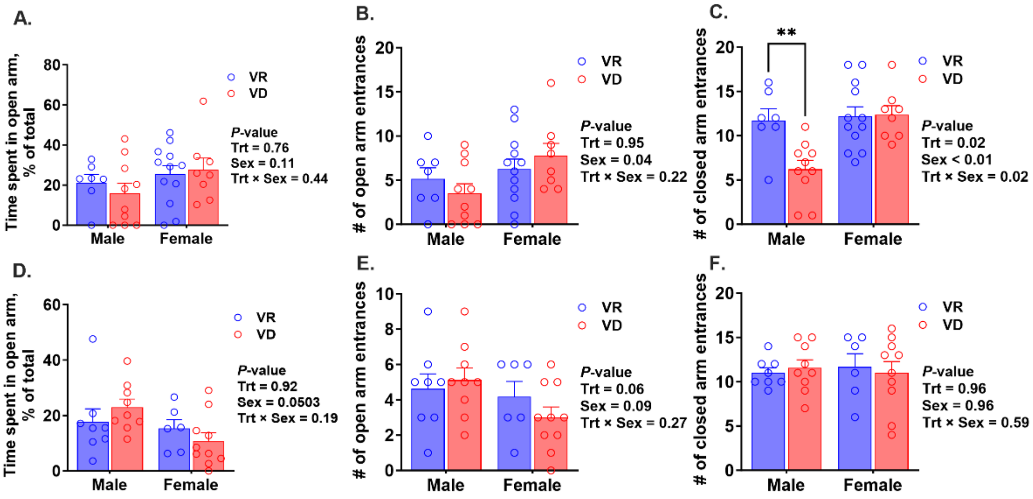

Figure S2. Effect of dietary vitamin A deficiency on anxiety parameters in offspring via an elevated plus maze. The 1st gestation of mice (G1, N= 7-12 per sex and treatment) and the percentage of duration spent in open arm (A), the number of entrances toward the open arm (B), and the number of entrances toward the closed arm (C). The 2nd gestation of mice (G1, N= 6-10 per sex and treatment) and the percentage of duration spent in the open arm (A), the number of entrances toward the open arm (E), and the number of entrances toward the closed arm (F). VR, vitamin A-adequate diet; VD, vitamin A-free diet. Data present LS means ± SEM. ⁎ P < 0.05, ⁎⁎ P < 0.01, ⁎⁎⁎ P < 0.001, and ⁎⁎⁎⁎ P < 0.0001.

Pre- and postnatal vitamin A deficiency impairs motor skills without affecting trace mineral status in young mice

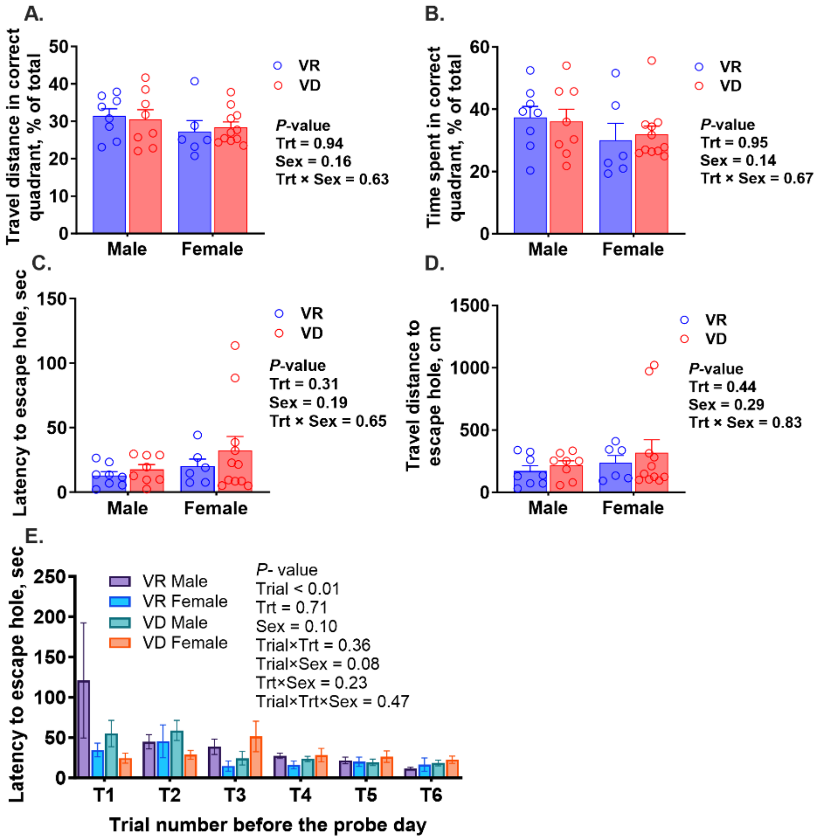

Figure S3. Effect of dietary vitamin A deficiency on spatial cognition. The percent distance traveled in the correct quadrant of where the escape hole is located (A), the percent of time spent of the total duration of the time on the Barnes Maze in the correct quadrant where the escape hole is located (B), the amount of time toward identifying the escape hole (C), and the distance traveled before identifying the escape hole (D). Latency to escape hole over several trials separated by sex and treatment (E). VR, vitamin A-adequate diet; VD, vitamin A-free diet. Data present LS means ± SEM. ∗ $P < 0.05$, ∗∗ $P < 0.01$, ∗∗∗ $P < 0.001$, and ∗∗∗∗ $P < 0.0001$.
